# Molecular docking analysis of α2-containing GABAA receptors with benzimidazoles derivatives

**DOI:** 10.6026/97320630016611

**Published:** 2020-08-31

**Authors:** Abdellatif Bouayyadi, Aissam El Aliani, Yassine Kasmi, Ahmed Moussaif, Najia El Abbadi, Abdelhalim Mesfioui, El Mokhtar Essassi, Mohammed El Mzibri

**Affiliations:** 1 Division of Life Sciences, National Centre for Energy, Nuclear Sciences and Techniques (CNESTEN), Morocco; 2 Laboratory of Genetic, Endocrinology and Biotechnology–Faculty of Sciences, Ibn Tofaïl University, Morocco; 3 Moroccan Foundation for Advanced Sciences, Innovation and Research. Morocco

**Keywords:** Benzemidazole, GABAA, GABAA receptor, anxiety, docking

## Abstract

It is of interest to study the binding capacity of "3-[2-(2-Amino-1H-benzo[d]imidazol-1-yl)ethyl]-1,3-oxazolidin-2-one" (OXB2) with the active site of gamma-aminobutyric acid (GABA) located in the GABA type A receptor (GABAAR) in comparison with different GABAA subtypes. Optimal binding features were observed with the α2β2γ2 isoform (-8 kcal/mol). This is similar (-7.3 and -7.2 kcal/mol, respectively) for subtypes (α3β2γ2 and α1β2γ2). This implies that OXB2 binds preferentially to subtypes associated with anxiety (α2- and/or α3-containing receptors) linked molecules than with the subtype associated with sedation (α1-containing receptors). It is further noted that molecular dynamics simulation data of the complex (OXB2-GABAAR) shows adequate structural stability in aqueous environment. Moreover, relevant ADMET data is found adequate for further consideration.

## Background

There is increasing interest to molecules containing heterocyclic ring, constituting the basic skeleton for a wide variety of compounds with industrial and pharmacological activities [1-2]. Heterocyclic compounds are the major chemicals, representing more than 60% of organic compounds and playing an important role in many biochemical processes [3]. Benzodiazepines are the main heterocyclic compounds used in medical therapy. These classes of psychoactive drugs are widely used for treatment of psychotropic diseases, especially Generalized Anxiety Disorder (GAD) [4- 5]. Benzodiazepines are also known for their sedative and hypnotic properties [6-7], and also for their amnesic, muscle relaxant and sedative characteristics [8-9]. Benzodiazepines act allosterically to enhance the central γ-amino-butyric acid (GABA)-mediated neurotransmission at the GABAA receptor [10]. GABAA receptors are ionotropic receptors and ligand-gated ion channel. Generally, GABAA receptors are pentameric proteins composed of different subunits (α1-6, β1-3, γ1-3, δ, ε, π and θ), α subunit being the most important one determining the pharmacology of the Benzodiazepines binding site [11]. The major Benzodiazepines -sensitive GABAA receptor subtypes in the brain are α1βxγ2, α2βxγ2, α3βxγ2 and α5βxγ2 and their distribution in the brain shows distinct regional variations [11]. Benzodiazepines are non-selective drugs and interact with all GABAA subtypes with equivalent affinity and efficacy, and consequently exert their therapeutic actions by modulating the function of GABA at GABAA receptors containing α1, or α2, α3 or α5 subunit [12-13].

Interest was given to delineate which α-subunit-containing GABAA receptors subtypes are associated with particular aspects of the diverse pharmacology of nonselective benzodiazepines. The functional heterogeneity of GABAA receptor subtype was initially implied on the basis of regional differences in the expression of different α subunit containing GABAA receptors [14-15] along with the novel pharmacological profile of the α1-subtype preferring hypnotic benzodiazepines drugs [16]. The functions of different GABAA receptor populations have been further clarified by the use of transgenic mice as well as subtype-selective compounds [17-18]. Hence, it is widely accepted that α1-containing GABAA receptors play a role in the sedative properties of the nonselective benzodiazepines [17-19] and anxiolytic properties are mediated by α2 and/or α3 subtypes [18,20-22].

Thereafter, great efforts are made to develop new anxiolytic drugs devoid of the sedative properties associated with classical benzodiazepines. In this regards, some anxioselective benzodiazepines were developed with much reduced sedative liability but have a lower intrinsic efficacy than existing benzodiazepines and therefore a limited clinical utility [23-24]. Currently, most studies focus on the development of compounds with subtype-selective efficacy, able to bind to all four subtypes, but with higher efficacy to α2- and α3- as compared to α1- and α5-containing receptors [25].

Benzimidazoles are heterocyclic aromatic compounds with large biological effects. Some benzimidazoles derivatives have shown a strong efficacy to cure psychotic disorders. Of particular interest, these compounds showed a good affinity to GABAA receptor with a clear selectivity to α2 and α3 subunits [26-29]. Recently, we have developed a new benzimidazole compound, 3-[2-(2-Amino-1H-benzo[d]imidazol-1-yl)ethyl]-1,3-oxazolidin-2-one" (OXB2), with a potential antidepressant / anxiolytic activities [30-31]. Therefore, it is of interest to study the binding capacity of "3-[2-(2-Amino-1H-benzo[d]imidazol- 1-yl)ethyl]-1,3-oxazolidin-2-one" (OXB2), a newly synthesized and characterized Benzimidazole, on the active site of gamma-aminobutyric acid (GABA) located in the GABA type A receptor (GABAAR) to compare with different GABAA subtypes.

## Methodology

### Synthesis of compounds:

**Figure 1:**
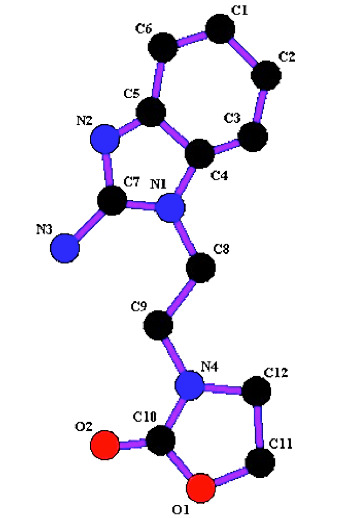
3-[2-(2-Amino-1H-benzo[d]imidazol-1-yl) ethyl]-1,3-oxazolidin-2-one (OXB2) represent in vivo effect on GABAA receptors generated by Ligplot

OXB2 is a new Benzimidazole derivative synthesized by a new method PTC (Phase-Transfer Catalysis) by combining family of Benzemidazoles and Oxazolines. The purity of the newly synthesized compound was verified by melting point and on (Thin Layer Chromatography) TLC and the structure was determined by various analytical techniques such as IR spectral studies and 1H NMR (Spectroscopy Nuclear Magnetic Resonance). In OXB2, the Benzimidazole ring is almost planar with the largest deviation from the mean plane being 0.039 (2) Å. However, the fused ring system is slightly folded at shared atoms with a dihedral angle of 3.4 (1)°. In contrast, the Oxazoline ring displays a twisted conformation on the adjacent carbon atoms. Moreover, the mean plane through the Oxazoline cycle makes a dihedral angle of 57.4 (2)° with the Benzimidazole ring. The molecules are linked together by two bifurcated N-H...O and C-H...N hydrogen bonds to form a three-dimensional network (Figure 1). There is also a weak C-H...π (benzene) interaction, which contributes to the stability of the crystal packing arrangement [30-31].

### Structure of GABAA receptor:

The crystal structure of the human's GABAAR was downloaded from RCSB database bearing the following crystallization specificities: Code PDB 4COF, which is the Crystal structure of a human gamma-aminobutyric acid receptor, the GABAAR-beta3 homopentamer published on 2014 by Miller, and Aricescu, by x-ray diffraction with resolution in order to 2.97 Å.R-Value Free: 0.226 and R-Value Work: 0.205.

### The Unit cell parameters were as:

Length [Å]: a = 174.10, b = 108.90 and c = 207.44.

Angles [°]: α = 90.00, β = 107.43 and γ = 90.00.

**Figure 2:**
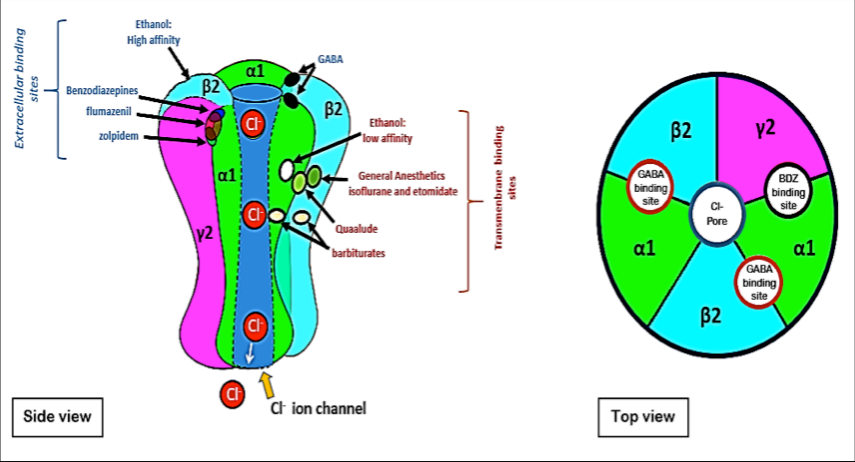
Structure of the GABAA receptor (side and top views)''' and position of the binding sites for different drugs.

As illustrated in Figure 2, the GABAA receptor is a molecular target for numerous CNS depressants including: benzodiazepines (e.g. librium, valium, medazolam), benzodiazepine- like hypnotics: (zolpidem, eszopiclone and zalepon which selectively bind to the α1 subunit of the GABAA receptor), Ethanol (at high and low affinity binding sites), barbiturates and anesthetics (e.g. isoflurane) [14-32]. As already mentioned, the GABAA receptors are composed by five subunits (2α, 2β, and 1γ). The GABA neurotransmitter bind in two sites (GABA site) localized between α and β subunit (top view). In other hand benzodiazepines like midazolam and benzodiazepine-like hypnotics like zolpidem bind in (BDZ site) localized between α and γ subunit (side view). Flumazenil (side view) is also BDZ receptor but has an antagonist characteristic, which can upset the effects of benzodiazepines and benzodiazepine-like hypnotics. The binding pocket is constructed from six regions, namely loops A-F. Experimental evidence reveals that the binding site in the GABAA receptor includes many residues (Table 1).

### Molecular Docking:

Molecular docking was used to evaluate the affinity of OXB2 to link to GABA (β2/α1, β2/α2, β2/α3) sites. The docking was performed on Autodock vina. The resulting structures were visualized using Chimera USCF [33] and PyMol [34], and 2D bond by LigPlus and Discovery Studio Visualization [35].

### Pharmacokinetic study:

ADME-Tox (absorption, distribution, metabolism, elimination and toxicity) profile evaluation is widely used to evaluate the potential pharmacokinetic characteristics of chemical compounds describing the different processes followed by the chemical after administration. ADME-Tox properties of OXB2 were studied using Pre-ADME and ADMET-Sar server [36]. The interactions between OXB2 and blood proteins were assessed by 3D-QSAR model.

### Molecular dynamics:

The molecular dynamics simulation has been carried out by GROMOS software using the server MDWeb [A] and gromacs [b]. The simulation was done by AMBER99SB Force Field and the following parameters: Time (ns) 10 and 50, Δt (ps) 0.1, Output Frequency (steps) 100, Force Constant (Kcal/mol*Å^2^) 40, Distance between Alpha Carbon Atoms (Å)3.0. The mutations showed in the alignment results were investigated in MD to study their effects on the structure of the protein.

## Results and Discussion

During last decades, pharmaceutical research has known a great evolution at both conceptual and methodological levels, using new technologies and innovative approaches. Bioinformatics and Cheminformatics tools have a special place in the process of valuing new synthetic components with cost and time gaining. In this study, bioinformatics tools were sued to evaluate the docking characteristics of OXB2, a newly synthesized molecule, on GABAA (β2/α1, β2/α2, β2/α3) receptors, to evaluate the molecular dynamic of this link and to assess the ADEM-Tox profile of OXB2.

GABAA (2α2, 2β2, and 1γ2) and GABAA (2α3, 2β2, and 1γ2) were modeled using I-TASSER server, using GABAA (2α1, 2β2, and 1γ2) (Id: 4COF) as a template. The total energy variation showed that for the 3 GABAA isoforms, the energy was around -7/-8 Kcal/mol, indicating that OXB2 is able to link to both GABAA receptors. Specific energy liaison of OXB2 to the 3 GABAA isoforms is reported in Table 2 and showed that the high energy score was obtained with the isoform GABAA (α2) giving a score of -8 kcal/mol.

**Figure 3:**
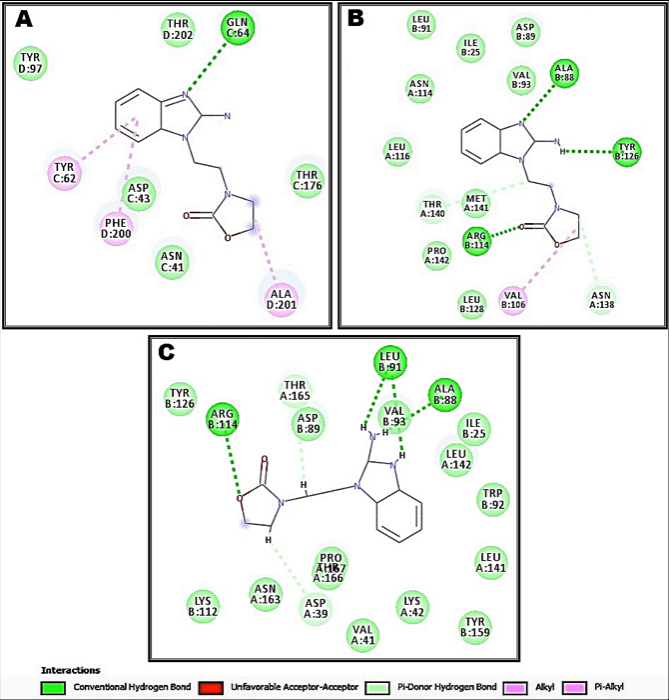
Schematic representation of interactions observed between OXB2 and GABAA (α1) *A*, GABAA (α2) *B* and GABAA (α3) *C* generated by discovery studio visualize

Interactions between OXB2 and GABAAR (α1), GABAAR (α2) and GABAAR (α3) are represented in Figure 3. Overall, OXB2 component forms fewer bonds with the active sites and all formed bonds are non-covalent type. The absence of covalent bonds can be explained by compatibility of the shape of the OXB2 with the active site. The Table 3 shows the residues involved in binding with the ligand and three isoforms of alpha subunit GABAA receptor. Eleven amino acids GLN64, PHE200, TYR62, ALA201, ALA88, TYR126, ARG114, VAL106, ARG114, LEU91 and ALA88 residues of template are involved in interaction.

The proposed binding mode OXB2 revealed an affinity value of -7.2 kcal/mol with the isoforms α1. The N-atoms of OXB2 interacted with active sites of GABAA (α1) by forming H-bond with GLN64 at distances of 2.69996 Å. Also, Pi-Alkyl type of interaction observed between aromatic rings and TYR62 and PHE200 with distances 4.34717 Å and 4.8423 Å respectively. Alkyl type of interaction was observed between (ALA201) and "carbon 11" of the ligand with bond lengths of 3.8367 Å (Figure 3A). In GABAA (α2) isoform, the proposed binding mode OXB2 revealed an affinity value of -8 kcal/mol. OXB2 interact with many amino acids residues by forming hydrogen bonds with ALA88 (3.35142Å), TYR126 (2.46278Å), ARG114 (2.75004Å), THR140 (3.40151Å) and ASN138 (4.97569Å). Also Alkyl type of interaction was observed between VAL 106 and carbon 12 of the ligand with bond lengths of 3.8367 Å (Figure 3B). Otherwise, OXB2 revealed an affinity value of -7.3 kcal/mol with the isoforms of GABAA (α3). Exclusively, H-bond type of interaction was indicated with different amino acids. LEU91 forms two bonds with NH- OXB2 with bond lengths of 2.61522 Å and 2.46548 Å. Also ARG114, THR 165, ASP39 and ALA88 form the same type of bond with OXB2 with distance 2.26162 Å, 2.82758 Å, 3.04107 Å and 2.38929 Å respectively (Figure 3C).

The non-covalent bonds established between the chemical compound and GABAA receptors are of particular interest to favor the placement of the proper ligand at the active site with competition and reversibility, whereas covalent bonds are highly stable and mostly associated with irreversible effects [37]. The liaison between OXB2 and GABAA receptors exhibited an endothermic reaction, which thermodynamically favors the good orientation of the compound in the system due to the increase in the enthalpy effect according to the law of Internal Energy [38]. The increase in Van Der Waals (VdW) energy is an obvious result as the new components are characterized by the presence of nitrogen atom and core aromatics of 5, making the attractive effect of the components more significant [39].

It's widely accepted that knowledge at an atomic level of the structural and dynamic aspects of organized systems is particularly important for better understanding the functions of these complex molecular structures. In many cases, obtaining the microscopic details by conventional experimental techniques proves impossible. However, the true explosion of the computerized means initiated for about ten years, and the development of efficient algorithms, make possible the study of supra molecular assemblies of increasing complexity by the methods of theoretical chemistry [40].

**Figure 4:**
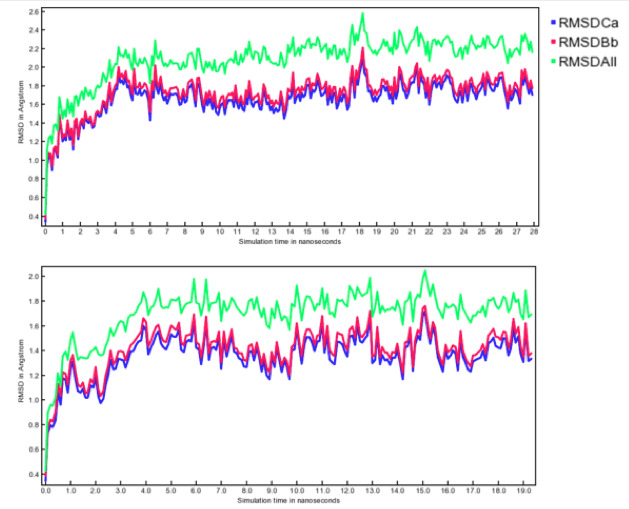
Molecular dynamic results

The complexes obtained by molecular docking were submitted to a simulation of 20 ns (Figure 4). Molecular dynamic results show stability of protein-ligand complex, characterized by thermal stability during the simulation conditions. The fluctuation of the protein complex is more stable for both complex and proteins; however the binding energy is more suitable for the complex than the protein alone. The simulations are done in a constant pressure system for the different cell dimension, which allowed having a prototypical simulation of the cellular activity during the whole dynamic simulation period. The energies of bonds, partially and VdW are very close, which lead to a high Van der Waals energy, just like a large number of hydrogen bonds since they pull the atoms closer than their normal Van der Waals contact distance.

**Figure 5:**
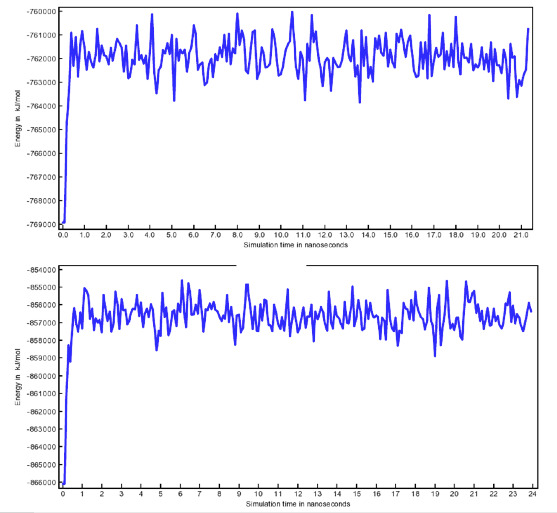
Total energy of systems

The total potential energies were calculated for each snapshot (Figure 5) and showed a fluctuation of about 1000 kcal / mol (about 0.5% of the total potential energy), indicating the stabilization of all the systems in MD simulations. In addition, the potential energies of complex models for each ligand subtype were quite similar, suggesting that the influence of local mutations on potential energy could be neglected in MD simulations. RMSD values were further calculated for each snapshot to study the relative movement of the backbone atoms of the proteins and ligands. RMSD values fluctuated largely when whole protein structures were considered in the calculation. Most of the RMSD values were less than 2 Å, and some of them even reached 0.6 Å (the complex model), indicating that the receptors showed less significant structural changes during the simulations. Since most parts of the complexes are less rigid and stable, the fluctuation of the RMSD values is mainly due to the loop. Thus, the RMSD values, excluding the complex, the structures were recalculated in Figure 4. The new RMSD values were generally less than 6 Å, demonstrating that the high RMSD values of the full-length receptors were attributed to the high flexibility of loops telling the active site.

A high value of factor B indicates more flexibility, while a low value of factor B indicates more stability. The helices had very low B-factor values, but the loops had moderate or high B-factor values, indicating large conformational changes in the loop regions during the MD simulations. These results were consistent with the inference of the RMSD values and explained that the high RMSD value of the complex was caused by a major conformational change, such as rotation. Although the flexibility of the loop has decreased the stability of the system, it would not affect inter-complex interactions because the loop was located far from the link interface. Thus, the reliability of further analysis can be guaranteed. ΔEvdW and ΔEelec oppose binding, but ΔGGB enhances binding to the complex by switching from CP to AP ligand, while ΔEvdW and ΔEelec improve binding. The sum of AEvdW and AEelec could overcome the term AGBG and cause the net link change. Decomposition analysis of binding energies In order to explore how mutations influence binding energies, binding energies are decomposed into each residue.

The pharmacodynamics and pharmacokinetic of newly synthesized drugs are of a great interest to evaluate the target and the undesirable effects and to appreciate the metabolization, bio-distribution, elimination and toxicity of the drug and its derivatives. In this study, the ADME-Tox profile of OXB2 was evaluated and results are reported in Table 4. In ADME-Tox analysis, the main parameter is the characterization of blood-brain barrier (BBB), evaluating the ability of drugs to cross this barrier and go insight the brain [41]. The role of BBB is to maintain brain homeostasis and to protect nerve tissue from circulating blood microorganisms, toxins, cellular factors and humoral immune system [42]. However, the presence of BBB prevents the treatment of many diseases of the central nervous system, and therefore in the perspective of psychotropic diseases therapy, all potential drugs have to cross the BBB and link to the target sites. ADME-Tox results showed that BBB permeability index was 0.554267, considered as medium to low [43], suggesting that OXB2 is able to cross the BBB and acts on GABAA receptors as target sites. Other important pharmacokinetic parameters were also predicted by Pre-ADME and ADMET-Sar and showed that OXB2 exhibited no AMES mutagenic and carcinogenic effects by Ames assay and possessed better human intestinal absorption. OXB2 had also Middle Caco2 permeability had a well human intestinal absorption. Predictive results showed that OXB2 weakly bounds to Plasma protein binding (PPB) and had lower MDCK permeability. These results suggest that OXB2 ligand has adequate pharmacokinetic characteristics and could be a promising candidate to be used as a drug.

## Conclusion

We document the molecular docking analysis of α2-containing GABAA receptors with a benzimidazole derivative for further consideration.

## Declaration on Publication Ethics:

The authors state that they adhere with COPE guidelines on publishing ethics as described elsewhere at https://publicationethics.org/. The authors also undertake that they are not associated with any other third party (governmental or non-governmental agencies) linking with any form of unethical issues connecting to this publication. The authors also declare that they are not withholding any information that is misleading to the publisher in regard to this article.

The authors are responsible for the content of this article. The Editorial and the publisher has taken reasonable steps to check the content of the article with reference to publishing ethics with adequate peer reviews deposited at PUBLONS.

## Figures and Tables

**Table 1:** amino acids and their characteristics in the binding site of GABAA

Amino acids characteristics	Noun and position
Aromatic (Alpha and beta subunit)	α1Phe64,β2Tyr62,β2Tyr97 andβ2Tyr205
Hydroxylated (Alpha and beta subunit)	α1Ser68,β2Thr160,β2Thr202,β2Ser204 andβ2Ser209
Charged (Alpha and beta subunit)	α1Arg120, α1Asp183, α1Arg66 andβ2Arg207

**Table 2:** Docking results of OXB2 with GABAA isoforms (α1, α2 and α3)

GABA (αn)	Docking Score
α1*	-7.2 kcal/mol
α2**	-8.0 kcal/mol
α3**	-7.3 kcal/mol
*Crystal structure **Modeled structure

**Table 3:** GABAA active site residues involved in docking interactions with the compounds

α1	α2	α3
GLN64	ALA88	ARG114
PHE200	TYR126	LEU91
TYR62	ARG114	ALA88
ALA201	VAL106	LEU91
	ASN138	ASP39
	THR140	

**Table 4:** ADMET prediction of OXB2

Parameter	Value /Predictive result	Parameter	Value /Predictive result
Ames mutatest	Negative	HIA	96.623102
SK log S pure	-1.9298	CYP3A4 substrate	Weakly
SK log S buffer	-1.59987	CYP3A4 inhibition	Non
SK log P value	1.15268	CYP2D6 substrate	Non
SK log D value	1.15268	CYP2D6 inhibition	Non
Skin Permeability	-4.1626	CYP2C9 inhibition	Non
Pure water solubility mg/l	2894.73	CYP2C19 inhibition	Non
Plasma Protein Binding	52.137421	Caco2	19.5815
Pgp inhibition	Non	Buffer solubility mg/l	6187.83
MDCK	17.4024	BBB	0.554267
BBB (Blood Brain Barrier): High absorption CNS >2.0, Middle absorption CNS 2.0–0.1, Low absorption CNS <0.1 Caco2: High permeability >70, Middle permeability 4–70, Low permeability >4 HIA (Human Intestinal Absorbance): Well absorbed compounds 70–100%, moderately absorbed compounds 20–70%, Poorly absorbed compounds 0–20%. PPB (Plasma Protein Binding): Strongly Bound >90%, Weakly Bound <90%, MDCK: Higher permeability >500, Medium Permeability 25–500, lower permeability >25.
